# The impact of lymphedema on health-related quality of life up to 10 years after breast cancer treatment

**DOI:** 10.1038/s41523-021-00276-y

**Published:** 2021-06-01

**Authors:** Mads G. Jørgensen, Navid M. Toyserkani, Frederik G. Hansen, Anette Bygum, Jens A. Sørensen

**Affiliations:** 1 Department of Plastic Surgery, Research Unit for Plastic Surgery, Odense University Hospital, Odense, Denmark; 2 Clinical Institute, University of Southern Denmark, Odense, Denmark; 3 OPEN, Open Patient Data Explorative Network, Odense University Hospital, Odense, Denmark; 4 Department of Plastic Surgery and Burns Treatment, Rigshospitalet, Copenhagen, Denmark; 5 Department of Clinical Genetics, Odense University Hospital, Odense, Denmark

**Keywords:** Pain, Breast cancer, Epidemiology

## Abstract

The impact of breast cancer-related lymphedema (BCRL) on long-term quality of life is unknown. The aim of this study was to investigate the impact of BCRL on health-related quality of life (HRQoL) up to 10 years after breast cancer treatment. This regional population-based study enrolled patients treated for breast cancer with axillary lymph node dissection between January 1st 2007 and December 31th 2017. Follow up and assessments of the included patients were conducted between January 2019 and May 2020. The study outcome was HRQoL, evaluated with the Lymphedema Functioning, Disability and Health Questionnaire, the Disabilities of the Arm, Shoulder and Hand Questionnaire and the Short Form (36) Health Survey Questionnaire. Multivariate linear logistic regression models adjusted for confounders provided mean score differences (MDs) with 95% confidence intervals in each HRQoL scale and item. This study enrolled 244 patients with BCRL and 823 patients without BCRL. Patients with BCRL had significantly poorer HRQoL than patients without BCRL in 16 out of 18 HRQoL subscales, for example, in physical function (MDs 27, 95%CI: 24; 30), mental health (MDs 24, 95%CI: 21; 27) and social role functioning (MDs 20, 95%CI: 17; 23). Age, BMI, BCRL severity, hand and dominant arm affection had only minor impact on HRQoL (MDs < 5), suggesting a high degree of inter-individual variation in coping with lymphedema. This study showed that BCRL is associated with long-term impairments in HRQoL, especially affecting the physical and psychosocial domains. Surprisingly, BCRL diagnosis rather than clinical severity drove the largest impairments in HRQoL.

## Introduction

Breast cancer is one of the most common forms of cancer with a yearly incidence of >1.5 million worldwide^1^. Breast cancer-related arm lymphedema (BCRL) is one of the most frequent and feared side effects to breast cancer treatment affecting >1 in 3 patients treated with axillary lymph node dissection (ALND)^2,3^. Externally, BCRL is characterized as a swelling of the arm, however patients frequently report a myriad of related symptoms, such as heaviness, tightness and pain in the arm, which can vary in intensity and is often discordant to objective severity^4–6^. The primary treatment for BCRL is conservative physiotherapy with complete decongestive therapy and microsurgery in selected cases^7–9^. Overall BCRL symptomatology is poorly understood, which complicates the diagnosis of BCRL and treatment expectations. Therefore, it is paramount to distinguish the arm morbidity and psychosocial impact of BCRL from those sequelae expected to arise following surgical and adjuvant breast cancer treatment itself.

Accordingly, this study was performed, as there were no published data on the long-term impact of BCRL on health-related quality of life (HRQoL) and which variables contribute to a higher degree of disability in BCRL patients.

## Results

### Data acquisition and demographics

**Fig. 1:**
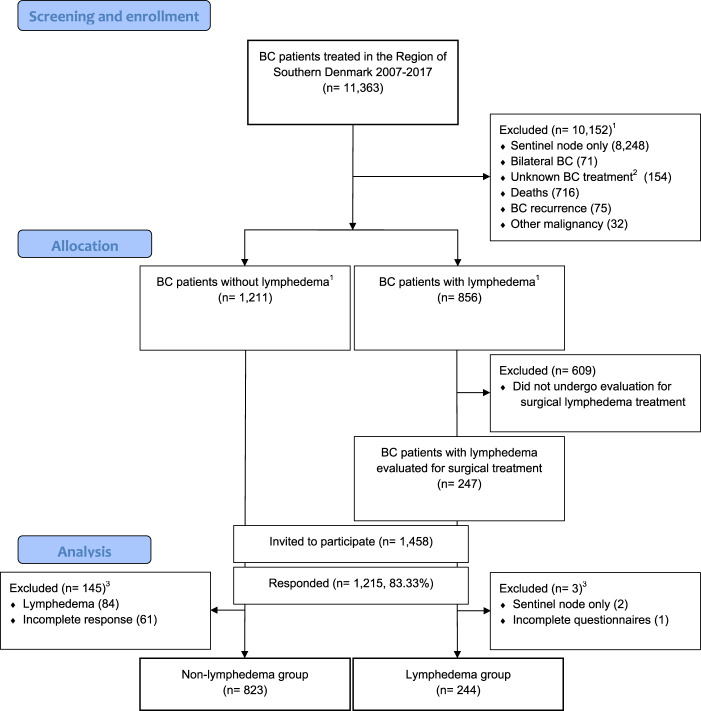
Flowchart of the included patients. This figure shows the flowchart of patients treated for breast cancer in the region of southern Denmark. 1 = Exclusion and allocation based on procedure-, treatment- and diagnostic codes. 2 = outside or unknown Danish Breast Cancer Group treatment protocol. 3 = Exclusion of patients based on chart reviews and questionnaire responses. BC breast cancer.

We included 1067 breast cancer patients with a history of axillary lymph node dissection and a mean follow up time of 7.95 ± 3.67 years since breast cancer treatment. Of the 1067 included patients, 244 had BCRL (see Fig. 1 for flowchart and Supplementary Table 1).

There were some expected differences between patients with and without BCRL given known risk factors for BCRL^2^. Patients with BCRL were slightly younger (*p* < 0.001) and slightly more overweight (*p* < 0.001), than patients without BCRL (Table 1). More patients with BCRL were treated with mastectomy (*p* < 0.001), radiation (*p* < 0.001) and chemotherapy (*p* < 0.001) and had more metastatic lymph nodes (*p* < 0.05) compared to patients without BCRL. In addition, more patients with BCRL had a history of arm cellulitis compared to breast cancer patients without BCRL (*p* < 0.001). For patients with BCRL, the mean duration of BCRL was 5.80 ± 4.32years. The mean BCRL volume was 406.35 ± 323.60 mL corresponding to an 18.59 ± 13.92% increase in excess arm volume compared to the healthy arm. Compression sleeve, compression gauntlet, night bandage and compression pumps were used daily as monotherapy or in combination in 185 (75.82%) patients. Lymphedema affected the arm only in 129 (52.87%) patients and 115 (47.13%) patients had lymphedema affecting their hand as well. For clinical BCRL stage, 19 (7.79%) patients had subclinical stage 0, 47 (19.26%) patients had stage 1, 109 (44.67%) had stage 2A, 67 (27.46%) had stage 2B and 2 (0.83%) patients had stage 3 clinical BCRL.

**Table 1:** Demographics and clinical characteristics of the 1067 patients included in the study.

Patient characteristics	Total (*n* = 1067)	No Lymphedema (*n* = 823)	Lymphedema (*n* = 244)	Test statistics^a^
	No. (%) or mean ± SD	No. (%) or mean ± SD	No. (%) or mean ± SD	*p*-value
Female (y/n)	1064 (99.72%)	823 (100%)	241 (98.77%)	<0.001
Age (years)	64.35 ± 10.23	65.51 ± 9.99	59.73 ± 9.85	<0.001
Body mass index (kg/m^2^)	26.91 ± 8.93	26.62 ± 9.65	28.12 ± 4.76	<0.001
In relationship (y/n)	756 (70.92%)	579 (70.44%)	177 (72.54%)	n.s
Hypertension (y/n)	309 (28.99%)	227 (27.62%)	82 (33.61%)	n.s
Breast cancer treatment				
Chemotherapy (y/n)	738 (69.17%)	534 (64.88%)	204 (83.61%)	<0.001
Radiation therapy (y/n)	929 (87.07%)	699 (84.93%)	230 (94.26%)	<0.001
Endocrine therapy (y/n)	862 (80.79%)	664 (80.68%)	198 (81.15%)	n.s
Lymph nodes removed (no)	17.33 ± 6.37	17.19 ± 6.42	17.92 ± 6.12	n.s.
Metastatic lymph nodes (no)	2.78 ± 4.87	2.64 ± 4.82	3.43 ± 5.04	<0.05
Mastectomy (y/n)	408 (38.27%)	285 (34.63%)	123 (50.62%)	<0.001
Operated on dominant side (y/n)	545 (51.08%)	427 (51.88%)	118 (48.36%)	n.s
Previous arm infection (y/n)	137 (12.89%)	55 (6.69%)	82 (34.02%)	<0.001

This table shows the demographic and baseline characteristics of included patients stratified by lymphedema occourence.

*BMI* body mass index (kg/m^2^), *BC* breast cancer.

^a^Students *t*-test or chi-squared.

### Quality of life impact of BCRL

**Fig. 2:**
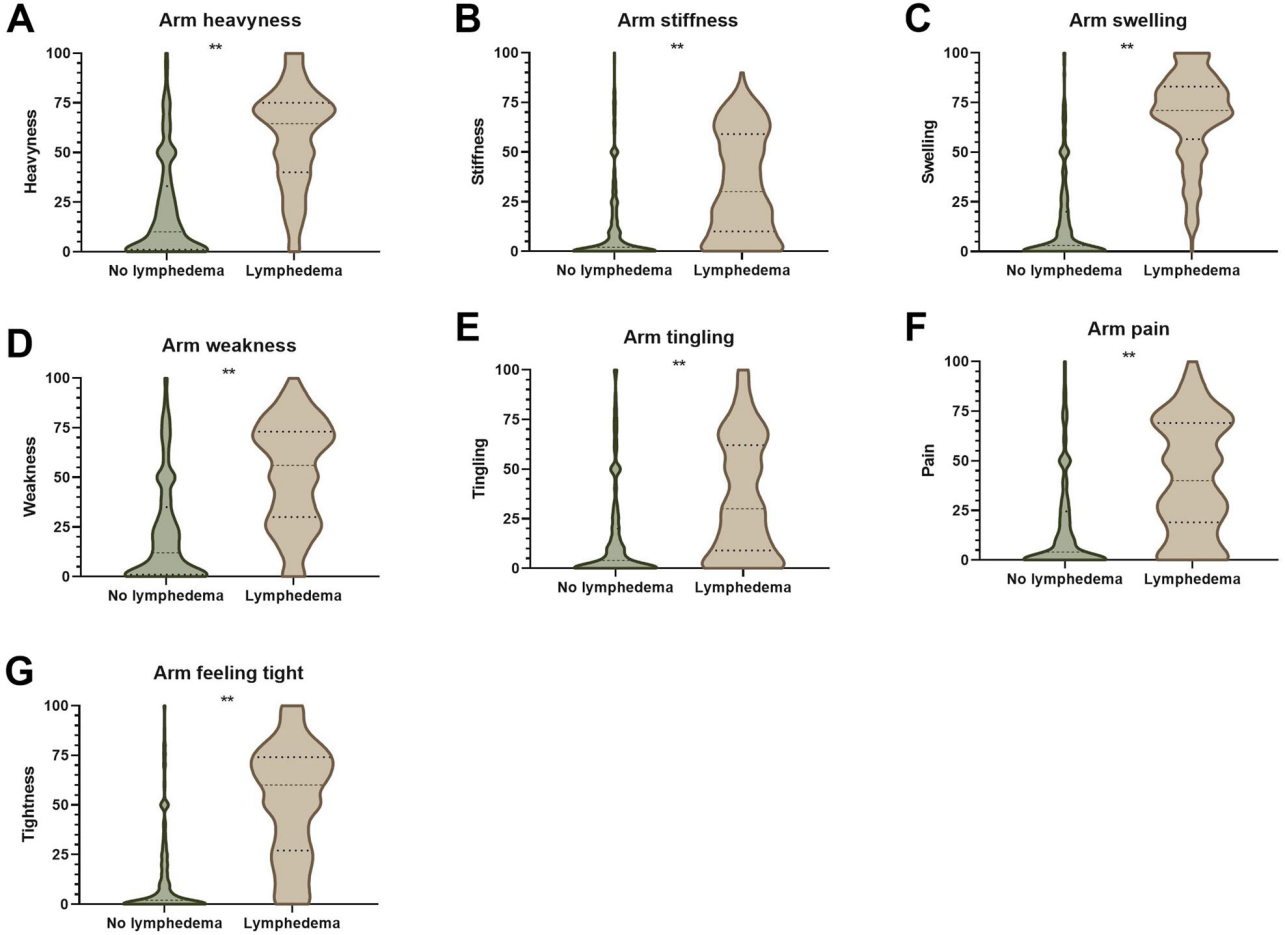
Patient reported symptoms of lymphedema. These violin plots show lymphedema symptoms reported by breast cancer patients treated with axillary lymph node dissection with and without lymphedema. Symptoms reported on a 0–100 numeric rating scale where 0 denotes no symptoms and 100 denote worst symptoms and compared using student’s *t*-test. Violin plot thickness denotes the probability density of each reported symptom. The height of the violin plots denotes the range of responses. Thin dashed line denotes the median and thick dashed lines denote the interquartile range. **A** Arm heaviness symptom. **B** Arm stiffness symptom. **C** Arm swelling symptom. **D** Arm weakness. **E** Arm tingling. **F** Arm pain. **G** Arm tightness. **p-value < 0.001.

Patients with BCRL reported worse feelings of arm heaviness (MDs 34.03, 95%CI: 30.50; 37.56, CS: large), stiffness (MDs 19.52, 95%CI: 16.39; 22.66, CS: moderate), swelling (MDs 52.50, 95%CI: 49.38; 55.62, CS: large), weakness (26.57, 95%CI: 22.90; 30.723, CS: large), tingling (MDs 17.90, 95%CI: 14.21; 21.69, CS: moderate), pain (MDs 23.13, 95%CI: 19.56; 26.71, CS: large) and tightness (MDs 37.76, 95%CI: 34.34; 41.18, CS: large) than patients without BCRL (Fig. 2A–G).

The BCRL group reported worse lymphedema-specific physical function (MDs 27.42, 95%CI: 24.58;30.28, CS: large) (Table 2 and Supplementary Table 2), generic mental health (MDs −23.82, 95%CI: −26.72;−20.93, CS: large), generic social role functioning (MDs −19.96, 95%CI:−23.05;−16.86, CS: moderate), impairment doing recreational activities (MDs 13.65 95%CI: 9.39;17.92, CS: moderate), impairment doing household activities (MDs 12.52 95%CI: 9.03;16.01, CS: moderate), worse lymphedema-specific mental functioning (MDs 11.20 95%CI: 8.19; 14.21, CS: moderate), life and social activities (MDs 10.32, 95%CI: 6.99; 13.65, CS: moderate) and mobility activities (MDs 9.98, 95%CI: 6.70; 13.26, CS: small), more impairment in physical role functioning (MDs −6.86, 95%CI:−12.46; −1.25, CS: small), general health perception (MDs −5.49, 95%CI: −8.64; −2.35, *p* < 0.05, CS: small), occupational impairment (MDs 5.66 95%CI: 2.44; 8.88, CS: small) and bodily pain (MDs 5.28 95%CI: 8.60; 1.97, CS: small) compared to patients without BCRL (Supplementary Fig. 1).

**Table 2:** Absolute health-related quality of life scores and adjusted mean score differences for patients with and without lymphedema.

Quality of life scales	Total (*n* = 1067)	No Lymphedema (*n* = 823)	Lymphedema (*n* = 244)	Adjusted linear regression^a^ [reference: no lymphedema]	
	Mean ± SD	Mean ± SD	Mean ± SD	MDs (95%CI)	*p*-value
Lymph-ICF total	21.34 ± 19.93	17.90 ± 19.18	34.46 ± 17.11	14.87 (12.18; 17.57)	<0.001
Physical function	24.61 ± 23.28	18.31 ± 19.93	48.65 ± 19.13	27.42 (24.58; 30.28)	<0.001
Mental function	14.36 ± 21.39	11.54 ± 19.76	24.83 ± 23.86	11.20 (8.19; 14.21)	<0.001
Household activity	21.59 ± 25.00	18.74 ± 24.70	32.36 ± 23.19	12.52 (9.03; 16.01)	<0.001
Mobility activity	22.82 ± 23.25	20.51 ± 23.49	31.55 ± 20.05	9.98 (6.70; 13.26)	<0.001
Life and social activity	20.38 ± 23.44	17.95 ± 23.74	29.50 ± 19.76	10.32 (6.99; 13.65)	<0.001
DASH	17.27 ± 15.91	15.47 ± 15.81	24.31 ± 14.33	7.83 (5.59; 10.07)	<0.001
Recreational (optional)	10.45 ± 16.60	8.36 ± 14.97	15.78 ± 19.21	13.65 (9.39; 17.92)	<0.001
Work (optional)	14.59 ± 19.63	11.70 ± 18.46	26.49 ± 19.90	5.66 (2.44; 8.88)	<0.001
SF-36 total	73.09 ± 18.94	74.99 ± 19.56	65.80 ± 14.19	−8.04 (−10.74; −5.35)	<0.001
Physical function	77.62 ± 20.99	77.97 ± 22.31	76.28 ± 14.85	−1.75 (−4.74; 1.24)	n.s.
Physical role functioning	70.20 ± 38.45	72.03 ± 38.37	63.46 ± 38.05	−6.86 (−12.46; −1.25)	<0.05
Emotional role functioning	78.45 ± 34.50	78.94 ± 34.55	76.68 ± 34.32	−2.00 (−7.05; 3.06)	n.s.
Vitality	64.25 ± 23.58	67.01 ± 24.18	53.75 ± 17.55	−10.51 (−13.83; −7.19)	<0.001
Mental health	75.23 ± 22.49	80.57 ± 18.32	54.95 ± 25.13	−23.82 (−26.72; −20.93)	<0.001
Social role functioning	83.82 ± 23.14	88.38 ± 20.99	66.59 ± 22.79	−19.96 (−23.05; −16.86)	<0.001
Bodily pain	77.20 ± 23.27	78.83 ± 23.71	71.08 ± 20.45	−5.28 (−8.60; −1.97)	<0.05
General health perception	66.78 ± 21.67	68.16 ± 22.89	61.48 ± 15.12	−5.49 (−8.64; −2.35)	<0.05

This table shows the quality of life of patients with and without lymphedema.

*MDs* mean score difference, *Lymph-ICF* Lymphoedema Functioning, Disability and Health Questionnaire, *DASH* Disabilities of the Arm, Shoulder and Hand Questionnaire. *SF-36* Short Form (36) Health Survey Questionnaire, *n.s*. not significant.

^a^Linear regression analysis adjusted for age, body mass index, relationship status, alcohol consumption, smoking consumption, arm dominance and time since breast cancer treatment.

### Inter-BCRL group differences in quality of life

Increased BCRL severity was associated with more severe feelings of arm heaviness (MDs pr. 10% increase 4.40, 95%CI: 2.04; 6.77, CS: small), stiffness (MDs pr. 10% increase 4.31, 95%CI: 1.78; 6.83, CS: small), swelling (MDs pr. 10% increase 6.18, 95%CI: 4.14; 8.22, CS: small) and tightness (MDs pr. 10% increase 5.77, 95%CI: 3.14; 8.39, CS: small, Supplementary Fig. 2A). Surprisingly, BCRL duration was not associated with improved or more severe lymphedema symptoms (Supplementary Fig. 2B). Increased BMI was also associated with more severe feelings of heaviness (MDs pr. unit 0.85, 95%CI: 0.21; 1.50, CS: small), pain (MDs pr. unit 0.93, 95%CI: 0.15; 1.70, CS: small) and tightness (MDs pr. unit 0.75, 95%CI: 0.03; 1.47, CS: small, Supplementary Fig. 2C). On the contrary, increasing age was associated with less feelings of heaviness (MDs per 10 years −6.39, 95%CI: −9.57; −3.21, CS: small), swelling (MDs per 10 years −4.09, 95%CI: −6.85; −1.34, CS: small) and weakness (MDs per 10 years −5.66, 95%CI: −9.19; −2.14, CS: small, Supplementary Fig. 2D).

Improvements in lymphedema-specific quality of life was associated with increased patient age (MDs per 10 years, −2.35, 95%CI: −4.63; −0.09, CS: small) and worsened with increasing BMI (MDs per unit 0.49, 95%CI: 0.03; 0.95, CS: small), BCRL size (MDs per 10% 1.93 95%CI: 0.24; 3.61, CS: small) and for patients with lymphedema involving the hand (MDs 5.45, 95%CI: 0.80; 10.11, CS: small, Table 3 and Supplementary Table 3). Physical functioning improvements were associated with patient age (MDs per 10 years −3.24, 95%CI: −5.76; −0.73, CS: small) and worsened with increase in BMI (MDs per unit 0.57, 95%CI: 0.06; 1.07, CS: small) and BCRL size (MDs per 10% 3.38, 95%CI: 1.52; 5.25, CS: small). Mental functioning improvements were associated with increasing patient age (MDs per 10 years −3.45, 95%CI: −6.72; −0.19, CS: small). Household activities impairment improved per increase in patient age (MDs per 10 years −3.31, 95%CI: −6.38; −0.25, CS: small). Household activities impairment were worsened by increase in BMI (MDs per unit 0.71, 95%CI: 0.09; 1.33, CS: small), BCRL size (MDs per 10% 1.93, 95%CI: 0.34; 4.21, CS: small) and for patients with BCRL affecting their dominant arm (MDs 5.04, 95%CI: 0.76; 10.83, CS: small) and hand affection (MDs 7.19, 95%CI: 0.91; 13.47, CS: small). Arm mobility was more impaired in patients with hand lymphedema (MDs 6.40, 95%CI: 0.84; 11.98, CS: small). Life and social activities impairment were more severe with increase in arm size (MDs per 10% 2.58, 95%CI: 0.61; 4.55, CS: small).

**Table 3:** Health-related quality of life mean difference scores within the lymphedema group.

Lymph-ICF	Lymph-ICF total			Physical function			Mental function			Household activities			Mobility activity			Life and social activity		
Variables	MDs	95%CI	*p*-value	MDs	95%CI	*p*-value	MDs	95%CI	*p*-value	MDs	95%CI	*p*-value	MDs	95%CI	*p*-value	MDs	95%CI	*p*-value
Age (per 10 years)	−2.35	−4.63; −0.09	<0.05	−3.24	−5.76; −0.73	<0.05	−3.45	−6.72; −0.19	<0.05	−3.31	−6.38; −0.25	<0.05	−1.10	−3.82; 1.62	n.s	−1.95	−4.62; 0.71	n.s
Body mass index (per 1 unit)	0.49	0.03; 0.95	<0.05	0.57	0.06; 1.07	<0.05	0.18	−0.48; 0.84	n.s	0.71	0.09; 1.33	<0.05	0.50	−0.06; 1.05	n.s	0.42	−0.12; 0.96	n.s
In relationship (y/n)	−2.28	−7.18; 2.61	n.s	−3.56	−8.94; 1.81	n.s	−4.51	−11.50; 2.49	n.s	−0.72	−7.34; 5.89	n.s	−1.33	−7.18; 4.52	n.s	−2.10	−7.79; 3.60	n.s
Alcohol (y/n)	−3.18	−8.22; 1.86	n.s	−1.37	−6.91; 4.12	n.s	−2.72	−9.93; 4.49	n.s	−0.88	−7.70; 5.94	n.s	−6.19	−12.21; 0.16	n.s	−2.68	−8.55; 3.18	n.s
Smoking (y/n)	−1.17	−8.75; 6.42	n.s	−2.85	−11.18; 5.48	n.s	−8.63	−19.47; 2.22	n.s	3.31	−6.94; 13.57	n.s	0.85	−8.21; 9.91	n.s	2.54	−6.28; 11.36	n.s
Lymphedema duration (per 1 year)	0.13	−0.42; 0.69	n.s	0.04	−0.57; 0.65	n.s	0.41	−0.39; 1.20	n.s	0.24	−0.51; 0.99	n.s	0.00	−0.66; 0.66	n.s	0.23	−0.42; 0.87	n.s
Lymphedema size (per 10%)	1.93	0.24; 3.61	<0.05	3.38	1.52; 5.25	<0.001	0.62	−1.81; 3.03	n.s	1.93	0.34; 4.21	<0.05	0.96	−1.07; 2.97	n.s	2.58	0.61; 4.55	<0.05
Lymphedema in dominant arm (y/n)	−1.25	−5.55; 3.04	n.s	−1.89	−6.60; 2.82	n.s	−3.15	−9.28; 2.98	n.s	5.04	0.76; 10.83	<0.05	−1.86	−6.98; 3.26	n.s	−2.79	−7.78; 2.19	n.s
Previous arm infection (y/n)	−0.36	−5.36; 4.65	n.s	−1.02	−6.51; 4.48	n.s	−1.16	−8.32; 6.00	n.s	1.84	−4.92; 8.60	n.s	−0.45	−6.43; 5.53	n.s	−0.66	−6.48; 5.16	n.s
Daily lymphedema therapy (y/n)	0.70	−4.49; 5.89	n.s	0.57	−5.18; 6.31	n.s	−0.63	−8.09; 6.82	n.s	2.76	−4.24; 9.76	n.s	0.51	−5.67; 6.72	n.s	1.09	−4.98; 7.16	n.s
Hand affected (y/n)	5.45	0.80; 10.11	<0.05	3.96	−1.20; 9.11	n.s	6.36	−0.33; 13.04	n.s	7.19	0.91; 13.47	<0.05	6.40	0.84; 11.98	<0.05	4.04	−1.42; 9.49	n.s

This table shows the association of clinical variables and health-related quality of life in patients with breast cancer-related lymphedema.

*MDs* mean score difference, *n.s* not significant.

Fewer patients in the working age with BCRL were working full time compared to patients without BCRL (48.23% vs. 58.58%, *p* < 0.05, Supplementary Fig. 3A). Patients with BCRL worked on average 2.7 h less per week than patients without BCRL (29.08 vs. 31.78 h per week, *p* < 0.05, Supplementary Fig. 3B). Interestingly, patients with BCRL reported more impairment doing desk jobs and physical jobs compared to patients without BCRL (MDs 9.19, 95%CI: 5.83; 12.55, CS: small and MDs 8.09, 95%CI: 2.94; 13.25, CS: small, respectively, Supplementary Fig. 3C). A surprising finding was that both groups of breast cancer patients reported more disability performing physical jobs compared to desk jobs (MDs 6.65, 95%CI: 0.38; 12.93, CS: small and MDs 6.79, 95%CI: 3.79–9.79, CS: small, respectively).

The LYMPH-ICF questionnaire correlated strongly with the DASH questionnaire (*R*^2^ = 0.64, Supplementary Fig. 4A). Both the LYMPH-ICF and DASH questionnaires correlated moderately with the SF-36 questionnaire (*R*^2^ = 0.44 and *R*^2^ = 0.52, Supplementary Fig. 4B, C). The residuals between the LYMPH-ICF, DASH, and SF-36 were unbiased with a heteroscedastic pattern along the *x*-axis (Supplementary Fig. 4D–F).

## Discussion

In this regional population-based cross-sectional study of patients treated with ALND for locoregional breast cancer, BCRL was found to be independently associated with impaired HRQoL up to 10 years postoperatively. Surprisingly, BCRL diagnosis, rather than clinical severity drove the largest impairment in HRQoL.

The main strengths of this study is its large sample size, use of registries, objective measurements and validated HRQoL instruments reducing information and recall bias. We had a high response rate (80%), warranting sufficient statistical power and clinically meaningful conclusions. We are aware that our study may have three limitations. First, the lack of preoperative HRQoL measurements could be a potential source of bias, as patients with and without BCRL may have had different HRQoL prior to breast cancer treatment. However, measuring HRQoL at the time of breast cancer treatment is ambiguous, as patients are emotionally affected by the breast cancer diagnosis^10,11^. The second is a possible selection bias in the study, as the BCRL group consisted of surgical candidates^12^. Thus, we cannot rule out that the BCRL group may experience more disability than BCRL patients actively living with BCRL without seeking surgical treatment. However, this is less of a concern, because the BCRL patients included in this study, had comparable breast cancer treatment paradigms (number of removed lymph nodes and rates of chemotherapy, radiation, endocrine, and mastectomy) and age as the reference BCRL patients not included in this study. Additionally, the BCRL patient group included in this study comprises a broad clinical spectrum of representative severities comparable to published reference BCRL patients^13,14^, and their HRQoL scores were strikingly similar to published HRQoL scores of the general BCRL population^15–18^. The third is a possible source of detection bias when identifying patients without BCRL in the study. It is notorious that the frequency of BCRL is dependent on the method for diagnosis^2,19^. In this study, we defined the diagnosis of lymphedema based on comprehensive registries, electronic patient chart reviews and patient questionnaires. We cannot rule out, that we may have underestimated the prevalence of BCRL, because we did not perform a clinical excess arm volume assessment of patients identified as not having BCRL. However, the potential risk of underestimation should be insignificant, given the uniform and free follow-up program for all breast cancer patients in Denmark and the completeness and validity of the Danish registries^20–22^. Access to electronic patient charts is only available at a regional level in Denmark, and therefore this study is based on regional rather than national data. However, there is no regional difference in breast cancer treatment across Denmark and all breast cancer centers follow DBCG treatment protocols^20^. Therefore, our findings should have great external validity.

The impact of BCRL on HRQoL is one of the most cited but poorly studied areas in BCRL research. A previous survey-only study from Denmark have reported a short-term prevalence of self-reported arm morbidity following sentinel lymph node biopsy and ALND. They found that prolonged time from surgery and young age was associated with higher risk of self-reported swelling^23^, and the incidence of self-reported swelling negatively affected emotional well-being and short-term adjustment to life after breast cancer^24^. Furthermore, smaller sized studies from Belgium, America and Australia showed that younger patients with BCRL reported worse HRQoL than older patients with BCRL, but that BCRL severity and duration did not affect short-term HRQoL^13,15,25^. The current studies have, however, been limited in their conclusions by a low number of patients with BCRL, unadjusted confounders and a lack of a comparison group to quantify the impact of BCRL. Additionally, the long-term impact of BCRL and affected HRQoL domains has previously not been investigated. In this study, we confirm the conclusions of smaller studies, and additionally quantify the impact and symptom burden of BCRL on HRQoL in a large dataset with long-term follow-up. We found that Llymphedema after breast cancer was associated with long-term impairment in HRQoL, especially in the physical and psychosocial domains. We further documented a large variation in coping with lymphedema, when adjusting for relevant confounders. Age, BMI, BCRL severity and affection of the hand and dominant arm all independently affected lymphedema symptoms and HRQoL. However, the MDs were small, and this together suggest a high degree of interpersonal variation in the perceived degree of disability and coping with BCRL. While our results are somewhat intuitive by nature, these conclusions have not previously been scientifically available and the poor understanding of BCRL symptomology leads to unmet patient expectations in treatment of BCRL^26^.

The reason for BCRL causing impaired HRQoL is likely to be multifactorial and can merely be speculated upon. One of the most clinically relevant findings, was the correlation between the LYMPH-ICF, DASH, and SF-36 responses. This suggest that patient’s lymphedema-specific impairments have significant impact on patient’s upper extremity mobility and handicap, which in turn translates into impaired overall quality of life. Patients with BCRL experience more arm swelling, weakness, tightness, heaviness, stiffness, pain and tingling compared to breast cancer patients without BCRL. The symptomatic sequelae of BCRL can have a negative psychological toll on the patient’s mental health, health perception and body image, limiting their engagement in social life and role functions. Swelling, and affection of the hand and dominant arm may decrease arm function and physically restrict patient’s engagement in recreation-, household-, and mobility activities.

The proportion of breast cancer patients surviving up to 10 years is increasing. The high incidence of BCRL following ALND signifies the increasingly important long-term impact of BCRL on HRQoL^2,3^. Health care professionals responsible for breast cancer patients must be aware of the negative consequences of BCRL on HRQoL. In the current age of de-escalating axillary surgery, the potential detrimental effect of BCRL should be weighed against the potential therapeutic benefit of elective ALND in early-stage breast cancer.

In conclusion, this study shows that BCRL is associated with impaired HRQoL outcomes up to 10 years after breast cancer treatment. We further found that BCRL diagnosis rather than severity drove the largest impairment in HRQoL. These results highlight the need for tailored rehabilitation and treatment programs to minimize the impact of BCRL on HRQoL. The results further encourage informed decision making regarding elective ALND, and the impact of BCRL and is especially relevant in the current era of de-escalating axillary surgery.

## Methods

### Study design and setting

This is a cross-sectional study of breast cancer patients with a history of axillary lymph node dissection with and without BCRL. This study was registered with the Danish Data Protection Agency (19/31965) and approved by The National Committee on Health Research Ethics (S-20180117) and The Danish Clinical Quality Program– National Clinical Registries (RKKP)/Danish Breast Cancer Cooperative Group (DBCG-2019-10-02). Informed consent was obtained from all patients involved in the study. The study was conducted in accordance with the Strengthening the Reporting of Observational studies in Epidemiology (STROBE) guidelines for cross-sectional studies^27^ and the criteria by Efficace et al. for reporting HRQoL outcomes^28^.

### Participants

The study participants comprises breast cancer patients treated with axillary lymph node dissection with and without BCRL. All patients were treated for breast cancer between 1st January 2007 and 31th December 2017 and follow up and assessments of all patients were conducted between January 2019 and May 2020. Baseline variables and data regarding breast cancer treatment were prospectively registered in the National Breast Cancer Registry from the Danish Breast Cancer Cooperative Group (DBCG), which was retrieved for this study. The DBCG include >95% of breast cancer patients in Denmark, and all breast cancer centers in Denmark follow the same treatment protocols regardless of geographical region^20^. This information included: (1) sex: female or male, (2) type of breast surgery: lumpectomy or mastectomy, (3) type of axillary procedure: sentinel lymph node biopsy or ALND, (4) total number of lymph nodes removed, (5) number of lymph nodes with metastases, (6) radiation therapy administered: yes or no, (7) chemotherapy administered: yes or no, and (8) endocrine therapy administered: yes or no (9) time of breast cancer treatment. Patients were excluded if they were treated with sentinel lymph node biopsy only, had bilateral breast cancer, un-protocolled breast cancer treatment, breast cancer recurrence or had had another malignant disease (apart from keratinocyte cancer). The following data was retrieved from all patients at assessment: (9) weight, (10) height, (11) relationship status, (12) comorbidities, (13) laterality of arm dominance: right or left, (14) laterality of breast cancer treatment: right or left, (15) regular weekly alcohol consumption: yes or no (15) regular weekly smoking: yes or no, (16) BCRL diagnosis, (17) previous arm cellulitis since breast cancer treatment, (18) current occupational status, (19) work title and description and (20) weekly working hours. Patients’ work title was then categorized into a binary variable if their work was primarily non-physical (e.g., office job, IT, administration) or primarily physical (e.g., manufacturing, nurse, gardener, hair-dresser, crafts).

Treatment codes for physiotherapeutic lymphedema treatment were used to confirm the diagnosis of BCRL and were retrieved from all hospitals in the region of southern Denmark or outside hospitals from referred BCRL patients. Identified patients with BCRL were further evaluated clinically in our Plastic Surgery subunit for surgical lymphedema treatment for: (21) duration of BCRL, (22) severity of BCRL using multiple circumference measurements to estimate limb volume^29,30^, (23) clinical BCRL stage^31^ and (24) current use of BCRL treatment. Incomplete or inconsistent information regarding BCRL diagnosis was supplemented by review of the electronic medical records.

### Health-related quality of life assessment

We measured the participants’ quality of life using 3 HRQoL instruments in REDCap^32^.

1. Lymphoedema Functioning, Disability and Health Questionnaire (LYMPH-ICF) is the most comprehensive, and accurate BCRL disease-specific questionnaire demonstrating high-content validity in the breast cancer population^33^. The LYMPH-ICF questionnaire has high reliability and was translated, validated and culturally adapted to the Danish population through international standards^34^. The questionnaire comprises five domains: lymphedema-specific physical function, mental function, household activities, mobility activities, and life and social activities. The questionnaire contains 29 statements that are scored on a numeric rating scale ranging from 0 to 100 by the patient. A total score can further be calculated using the mean of all domains.
2. Disabilities of the Arm, Shoulder and Hand Questionnaire (DASH), is a generic upper extremity functional questionnaire with high construct validity in the breast cancer population^35^. The DASH questionnaire has high reliability and was translated, validated and culturally adapted to the Danish population through international standards^36,37^. The questionnaire consists of 30 items evaluating upper limb-related activities and symptoms. Each item is scored from 0 to 100 for disability of the hand, arm and shoulder function or symptoms. A score of 0 means that the patient is not bothered at all and a score of 100 means the patient is bothered a lot. The questionnaire additionally contains two optional subscales regarding occupation and recreational activities.
3. The Short Form Health Survey Questionnaire (SF-36), is a generic health questionnaire, which provides a reliable and valid indication of general health status among the breast cancer population^38^. The SF-36 has high reliability and was translated, validated and culturally adapted to the Danish population through international standards^39–41^. The questionnaire consists of 36 items in 8 domains: vitality, physical function, bodily pain, general health perception, physical role function, emotional role function, social role function and mental health. Each scale is transformed into a 0–100 scale. A score of 100 means that the patient has no disability at all and a score of 0 means the patient has a lot of disability. A total score can further be calculated using the mean of all domains.

### Statistical methods

The baseline characteristics were described for patients with and without BCRL with means ± standard deviation (SD) for continuous variables and rounded frequencies (%) for categorical variables. Baseline characteristics were compared between patients with and without BCRL with an unpaired *t-*test or chi-squared test depending on data type. Multivariate linear regression models were used to calculate HRQoL mean score differences (MDs) with 95% confidence intervals (95%CI) between patients with and without BCRL. The analyses were adjusted for (1) age at assessment, (2) body mass index at assessment, (3) relationship status: currently in a relationship or not, (4) weekly alcohol consumption: yes or no, (5) weekly smoking: yes or no, (6) breast cancer treatment on the dominant arm side: yes or no and (7) time since breast cancer treatment. Multivariate linear regression models were used to analyze factors associated with significant MDs in lymphedema-specific HRQoL and symptoms within the BCRL group. The variables included in the models were (1) age at assessment, (2) body mass index at assessment, (3) relationship status: currently in a relationship or not, (4) weekly alcohol consumption: yes or no, (5) weekly smoking: yes or no, (6) time since lymphedema diagnosis, (7) excess lymphedema volume, (8) lymphedema in dominant arm: yes or no, (9) previous arm infection: yes or no, (10) daily use of conservative lymphedema therapy: yes or no and (11) lymphedema affecting the hand: yes or no. Baseline variables (age, body mass index, relationship status, alcohol and smoking) were chosen for the models a priori. Treatment and lymphedema-specific variables (breast cancer laterality, time since breast cancer, time since lymphedema, excess lymphedema volume, lymphedema laterality, arm infection episodes, use of lymphedema treatments and lymphedema involving the hand) were chosen due to their perceived significant impact on HRQoL in breast cancer patients^13,23,24,42^. As no established cutoff values were available to determine clinical significance, we considered MDs below 10 to be of minimal clinical significance, MDs of 10 to 20 points of moderate significance and 20 or more points to be of large clinical significance^43^. Correlation and residuals between LYMPH-ICF, DASH, and SF-36 responses were calculated using simple linear regression. STATA 15 (StataCorp. 2017. *Stata Statistical Software: Release 15*. College Station, TX: StataCorp LP) and GraphPad Prism (Version 8.00 for Windows, GraphPad Software, La Jolla California USA) were used for the statistical analysis and conducted with a two-tailed significance level of 0.05 and reported with 95%CI when applicable.

### Sample size

Sample size calculation was performed using STATA 15. As the risk of developing BCRL following ALND is ~33%, the final sample size allocation ratio between patients with and without BCRL was estimated to be 1:3. A total sample size of 856 study participants (213 with BCRL and 643 without BCRL) was designed to have an overall 80% power and a 5% significance level to detect a moderate clinical significance of 10% difference in LYMPH-ICF MDs between the BCRL and non-BCRL cohort given a common 45%SD based on published data^17,25,44^.

### Reporting summary

Further information on research design is available in the Nature Research Reporting Summary linked to this article.

## Supplementary information

Supplementary Information

Reporting Summary

## Data Availability

The data generated and analyzed during this study are described in the following data record: 10.6084/m9.figshare.14546208^45^. The data are contained in the STATA file ‘workfile_alldata.dta’ and the Excel spreadsheet ‘inclusion flowchart-key numbers.xlsx’. These data files are housed on institutional storage and are not publicly available for the following reason: data contain information that could compromise research participant privacy. However, the data can be made available upon reasonable request to Dr. Jørgensen or Dr. Sørensen, in accordance with Odense University Hospital and the Region of Southern Denmark’s data sharing policy as part of an external collaborative request. The individual-level data are not publicly available because of data privacy regulations and restrictions for use of such data, as stated in the study protocol and patient consent form.
