# Supplementary material for: The impact of lymphedema on health-related quality of life up to 10 years after breast cancer treatment
Source: NPJ Breast Cancer. 2021 Jun 1;7:70. doi: 10.1038/s41523-021-00276-y (PMC8169644; doi:10.1038/s41523-021-00276-y)
Supplement: Supplementary file 1 — Supplementary Information [file 41523_2021_276_MOESM1_ESM.pdf]

Supplementary Figure 1) Impact of lymphedema on quality of life

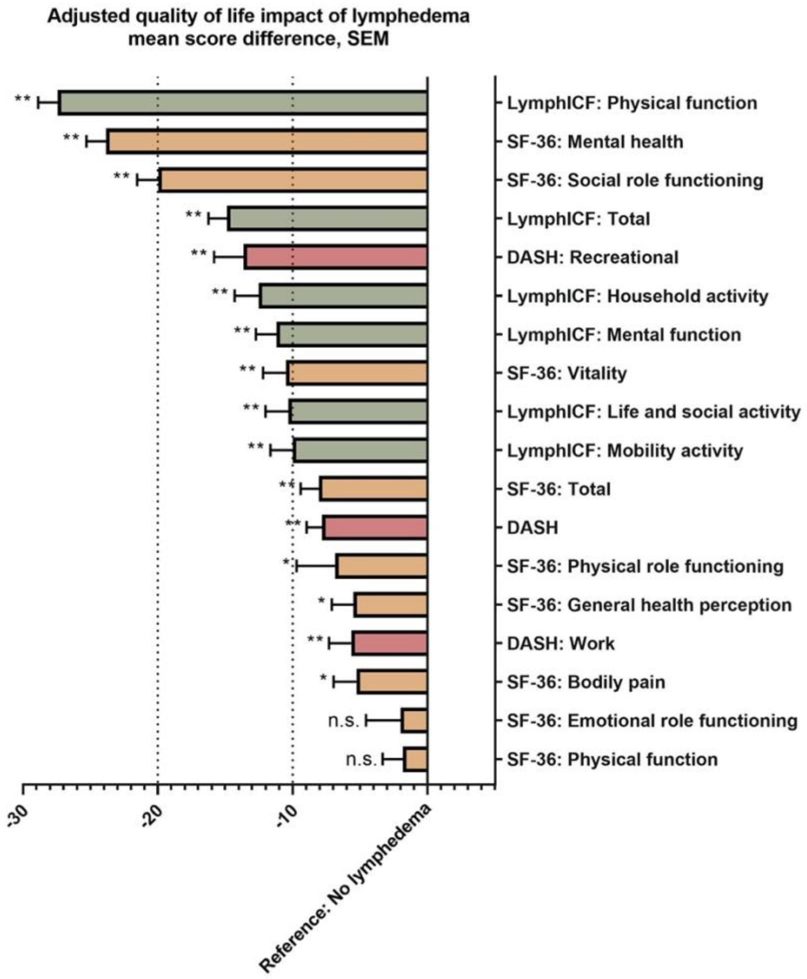

Supplementary Figure 1 legend: This figure shows the adjusted impact of lymphedema on quality of life using multiple logistic regressions. Mean score differences are adjusted for age, body mass index, arm dominance and time since breast cancer treatment. Negative values signify more impairment (worse) compared to patients without lymphedema. Scales ordered by mean magnitude of impairment. *= p-value <0.05. **= p-value <0.001. N.s. = not significant

Supplementary Figure 2) Correlation between lymphedema symptoms and lymphedema size, lymphedema duration, body mass index and age

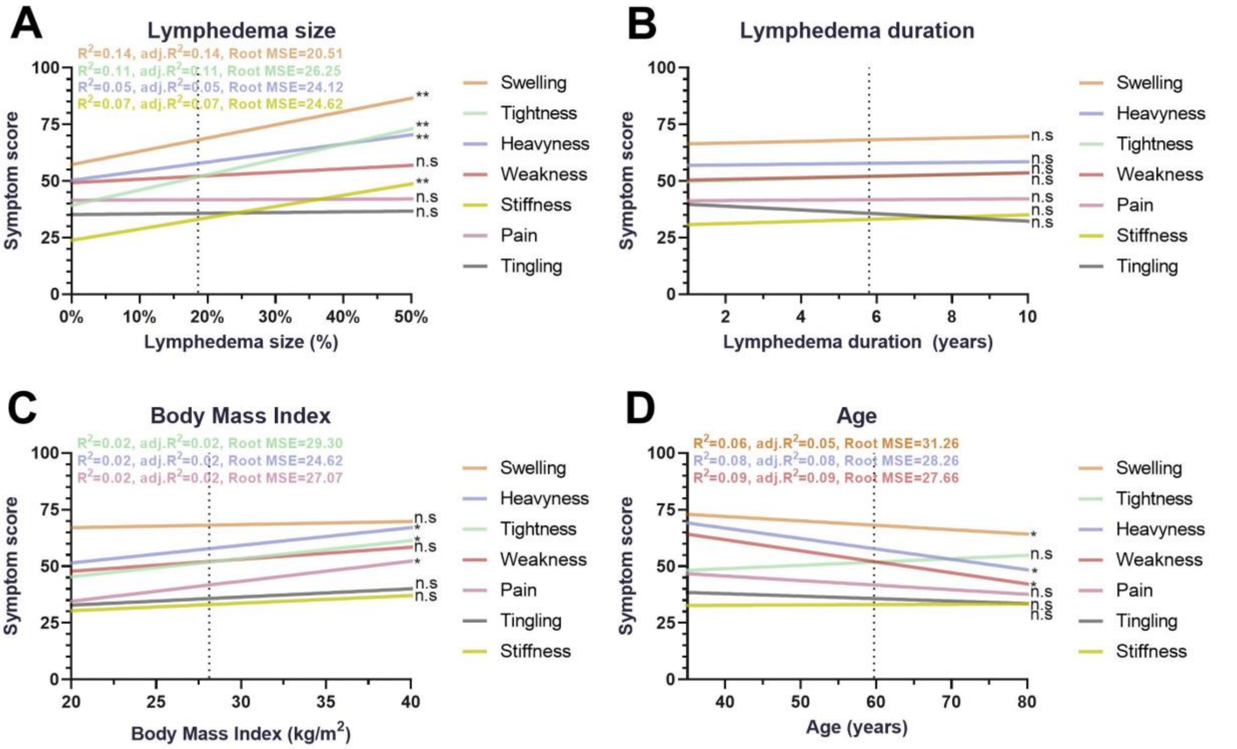

Supplementary Figure 2 legend: This figure shows the correlation between lymphedema symptoms and lymphedema size, lymphedema duration, body mass index and age for the lymphedema patients. Lymphedema symptoms include swelling, weakness, tightness, heaviness, stiffness, pain and tingling and were correlated to using linear regression. A) Correlation between lymphedema size and symptoms. Vertical dotted line denotes the mean lymphedema size for all lymphedema patients. B) Correlation between lymphedema duration and symptoms. Vertical dotted line denotes the mean lymphedema duration. C) Correlation between body mass index and lymphedema symptoms. Vertical dotted line denotes the mean body mass index. D) Correlation between age and lymphedema symptoms. Vertical dotted line denotes the mean age. Correlation performed using linear regression. Adj.R^2 = adjusted R^2. Root MSE = Root Mean Square Error of Estimate. * = p-value <0.05. ** = p-value <0.001. n.s = not significant.

Supplementary Figure 3) Impact of lymphedema on employment and work impairment

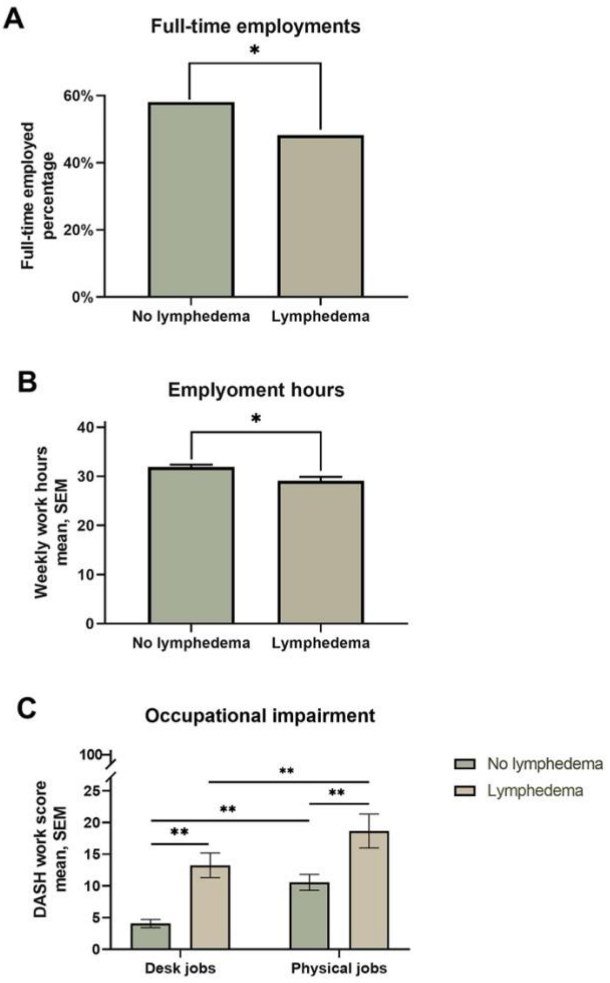

Supplementary Figure 3 legend: This figure shows the self-reported employments and occupation for patients with and without breast cancer-related lymphedema. A) Percentage of patients working full-time. B) Average employment hours per. C) Occupational impairment derived from the optional DASH work score stratified by lymphedema status and job description. Week. * = p-value <0.05. ** = p-value <0.001.

Supplementary Figure 4) Correlation between Lymph-ICF, DASH and SF-36 questionnaires

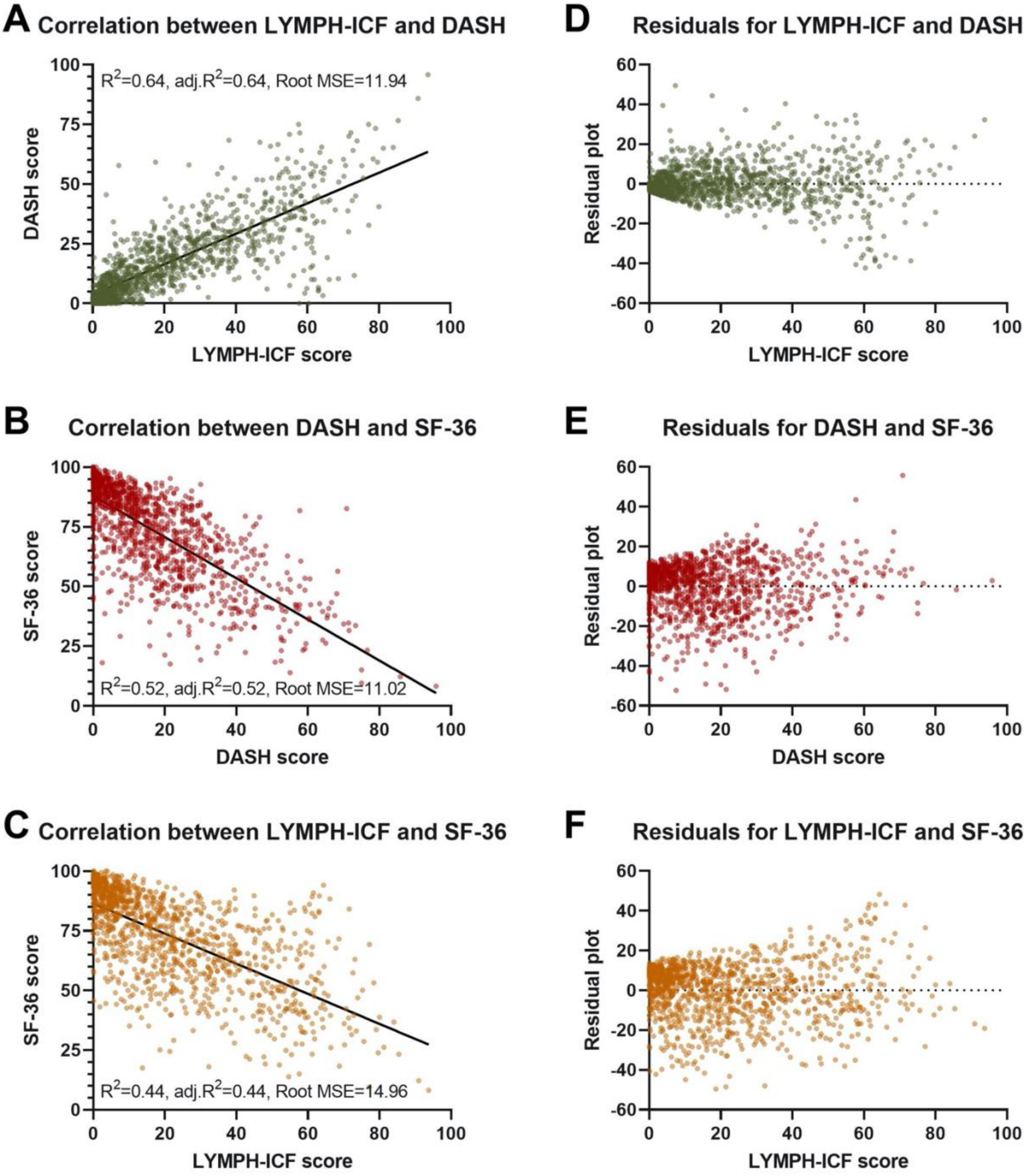

Supplementary Figure 4 legend: This figure shows the correlation and residuals between Lymph-ICF, DASH and SF-36 questionnaires. Lymph-ICF = Lymphoedema Functioning, Disability and Health Questionnaire. DASH = Disabilities of the Arm, Shoulder and Hand Questionnaire. SF-36 = The Short Form (36) Health Survey Questionnaire. A) Correlation between LYMPH-ICF and DASH. B) Residuals between LYMPH-ICF and DASH. C) Correlation between DASH and SF-36. D) Residuals between DASH and SF-36. E) Correlation between LYMPH-ICF SF-36. F) Residuals between LYMPH-ICF and SF-36. Correlation performed using linear regression. Adj.R^2=adjusted R^2. Root MSE = Root Mean Square Error of Estimate.

Supplementary Table 1) Overview of included and not included patients from this study

	**No lymphedema**		**Lymphedema**	
	**Not included (n =243)**	**Included (n =823)**	**Not included (n =609)**	**Included (n =244)[1]**
**Patient characteristics**	No. (%) or mean ±SD	No. (%) or mean ±SD	No. (%) or mean ±SD	No. (%) or mean ±SD
Age (years)	62.55±12.40	65.51±9.99	58.70±11.45	59.73±9.85
Chemotherapy (y/n)	146 (60.08%)	534 (64.88%)	470 (77.18%)	204 (83.61%)
Radiation therapy (y/n)	197 (81.07%)	699 (84.93%)	541 (88.83%)	230 (94.26%)
Endocrine therapy (y/n)	201 (82.72%)	664 (80.68%)	538 (82.21%)	198(81.15%)
Lymph nodes removed (no)	16.87±6.49	17.19±6.42	18.40±6.46	17.92±6.12
Metastatic lymph nodes (no)	2.51±6.33	2.64±4.82	4.07±5.4	3.43±5.04
Mastectomy (y/n)	98 (40.32%)	285 (34.63%)	295 (48.41%)	123 (50.62%)

Supplementary Table 1 legend: This table shows the available baseline characteristics from the patients included and not included in the study.

1: This number also includes patients living outside the Region of Southern Denmark that were referred to our clinic and included in this study

Supplementary Table 2) Supplementary information on the multivariate regression model presented in table 2

**Quality of life scales**	**R^2**	**Adjusted R^2**	**Root MSE**
**LymphICF total**	0.17	0.16	18.20
Physical function	0.31	0.30	19.39
Mental function	0.10	0.10	23.07
Household activity	0.10	0.09	23.70
Mobility activity	0.10	0.09	22.10
Life and social activity	0.09	0.09	22.51
**DASH**	0.11	0.10	15.08
Recreational (optional)	0.12	0.10	18.61
Work (optional)	0.10	0.08	15.90
**SF-36 total**	0.11	0.10	17.96
Physical function	0.12	0.11	19.81
Physical role functioning	0.05	0.05	37.65
Emotional role functioning	0.04	0.03	34.16
Vitality	0.11	0.10	22.33
Mental health	0.24	0.24	19.63
Social role functioning	0.19	0.19	20.83
Bodily pain	0.09	0.08	22.25
General health perception	0.05	0.04	21.28

Supplementary Table 2 legend: This table shows the R^2, adjusted R^2 and root MSE of the multivariate linear regression analyses in table 2. LymphICF = Lymphoedema Functioning, Disability and Health Questionnaire. DASH = Disabilities of the Arm, Shoulder and Hand Questionnaire. SF-36 = Short Form (36) Health Survey Questionnaire. Root MSE = Root Mean Square Error of Estimate.

Supplementary Table 3) Supplementary information on the multivariate regression model presented in table 3

	**Lymph-ICF**					
	**Total**	**Physical function**	**Mental function**	**Household activities**	**Mobility activity**	**Life and social activity**
**R^2**	0.11	0.13	0.06	0.12	0.08	0.08
**Adjusted R^2**	0.07	0.08	0.02	0.08	0.03	0.04
**Root MSE**	16.51	18.27	23.72	22.28	19.74	19.34

Supplementary Table 3 legend: This table shows the R^2, adjusted R^2 and root MSE of the multivariate linear regression analyses in table 3. LymphICF = Lymphoedema Functioning, Disability and Health Questionnaire. Root MSE = Root Mean Square Error of Estimate.
