# Supplementary material for: The impact of lymphedema on health-related quality of life up to 10 years after breast cancer treatment
Source: NPJ Breast Cancer. 2021 Jun 1;7:70. doi: 10.1038/s41523-021-00276-y (PMC8169644; doi:10.1038/s41523-021-00276-y)
Supplement: Supplementary file 2 — Reporting Summary [file 41523_2021_276_MOESM2_ESM.pdf]

nature research

Corresponding author(s): Mads Gustaf Jørgensen, MD
Department of Plastic Surgery, Odense University Hospital
J. B. Winsløws Vej 4
5000 Odense C
Mads.Gustaf.Jorgensen@rsyd.dk

Last updated by author(s): Feb 18, 2021

Reporting Summary

Nature Research wishes to improve the reproducibility of the work that we publish. This form provides structure for consistency and transparency in reporting. For further information on Nature Research policies, see our Editorial Policies and the Editorial Policy Checklist.

Statistics

For all statistical analyses, confirm that the following items are present in the figure legend, table legend, main text, or Methods section.

n/a	Confirmed	
☐	☒	The exact sample size (*n*) for each experimental group/condition, given as a discrete number and unit of measurement
☒	☐	A statement on whether measurements were taken from distinct samples or whether the same sample was measured repeatedly
☐	☒	The statistical test(s) used AND whether they are one- or two-sided<br>*Only common tests should be described solely by name; describe more complex techniques in the Methods section.*
☐	☒	A description of all covariates tested
☐	☒	A description of any assumptions or corrections, such as tests of normality and adjustment for multiple comparisons
☐	☒	A full description of the statistical parameters including central tendency (e.g. means) or other basic estimates (e.g. regression coefficient) AND variation (e.g. standard deviation) or associated estimates of uncertainty (e.g. confidence intervals)
☒	☐	For null hypothesis testing, the test statistic (e.g. *F*, *t*, *r*) with confidence intervals, effect sizes, degrees of freedom and *P* value noted<br>*Give P values as exact values whenever suitable.*
☒	☐	For Bayesian analysis, information on the choice of priors and Markov chain Monte Carlo settings
☒	☐	For hierarchical and complex designs, identification of the appropriate level for tests and full reporting of outcomes
☒	☐	Estimates of effect sizes (e.g. Cohen's *d*, Pearson's *r*), indicating how they were calculated

Our web collection on statistics for biologists contains articles on many of the points above.

Software and code

Policy information about availability of computer code

Data collection	Study data were collected and managed using REDCap electronic data capture tools hosted at Odense University Hospital[1,2] REDCap (Research Electronic Data Capture) is a secure, web-based software platform designed to support data capture for research studies, providing 1) an intuitive interface for validated data capture; 2) audit trails for tracking data manipulation and export procedures; 3) automated export procedures for seamless data downloads to common statistical packages; and 4) procedures for data integration and interoperability with external sources.<br><br>[1] PA Harris, R Taylor, R Thielke, J Payne, N Gonzalez, JG. Conde, Research electronic data capture (REDCap) – A metadata-driven methodology and workflow process for providing translational research informatics support, J Biomed Inform. 2009 Apr;42(2):377-81.<br><br>[2]PA Harris, R Taylor, BL Minor, V Elliott, M Fernandez, L O'Neal, L McLeod, G Delacqua, F Delacqua, J Kirby, SN Duda, REDCap Consortium, The REDCap consortium: Building an international community of software partners, J Biomed Inform. 2019 May 9 [doi: 10.1016/ j.jbi.2019.103208]
Data analysis	STATA 15 (StataCorp. 2017. Stata Statistical Software: Release 15. College Station, TX: StataCorp LP) and GraphPad Prism (Version 8.00 for Windows, GraphPad Software, La Jolla California USA)

For manuscripts utilizing custom algorithms or software that are central to the research but not yet described in published literature, software must be made available to editors and reviewers. We strongly encourage code deposition in a community repository (e.g. GitHub). See the Nature Research guidelines for submitting code & software for further information.

Data

Policy information about availability of data

All manuscripts must include a data availability statement. This statement should provide the following information, where applicable:

- Accession codes, unique identifiers, or web links for publicly available datasets
- A list of figures that have associated raw data
- A description of any restrictions on data availability

The datasets generated during and analysed during the current study are available from the corresponding author on reasonable request.

Field-specific reporting

Please select the one below that is the best fit for your research. If you are not sure, read the appropriate sections before making your selection.

☒ Life sciences ☐ Behavioural & social sciences ☐ Ecological, evolutionary & environmental sciences

For a reference copy of the document with all sections, see nature.com/documents/nr-reporting-summary-flat.pdf

Life sciences study design

All studies must disclose on these points even when the disclosure is negative.

Sample size	Sample size calculation was performed using STATA 15. As the risk of developing BCRL following ALND is approximately 33%, the final sample size allocation ratio between patients with and without BCRL was estimated to be 1:3. A total sample size of 856 study participants (213 with BCRL and 643 without BCRL) was designed to have an overall 80% power and a 5% significance level to detect a moderate clinical significance of 10% difference in LYMPH-ICF MDs between the BCRL and non-BCRL cohort given a common 45%SD based on published data
Data exclusions	145 patients were excluded in the non-BCRL cohort due to having BCRL or incomplete questionnaire responses.<br>3 patients were excluded in the BCRL cohort due to having been treated with ALND and incomplete questionnaire responses
Replication	The inclusion and allocation of patients were derived from valid and comprehensive cancer registries.
Randomization	Allocation into the BCRL and non-BCRL cohort was performed using comprehensive cancer registries, electronic patient chart reviews and questionnaires.
Blinding	Not relevant for the study.

Reporting for specific materials, systems and methods

We require information from authors about some types of materials, experimental systems and methods used in many studies. Here, indicate whether each material, system or method listed is relevant to your study. If you are not sure if a list item applies to your research, read the appropriate section before selecting a response.

Materials & experimental systems

n/a	Involved in the study	
☒	☐	Antibodies
☒	☐	Eukaryotic cell lines
☒	☐	Palaeontology and archaeology
☒	☐	Animals and other organisms
☐	☒	Human research participants
☒	☐	Clinical data
☒	☐	Dual use research of concern

Methods

n/a	Involved in the study	
☒	☐	ChIP-seq
☒	☐	Flow cytometry
☒	☐	MRI-based neuroimaging

Human research participants

Policy information about studies involving human research participants

Population characteristics	The study participants comprised of breast cancer patients treated with axillary lymph node dissection with and without BCRL. All patients were treated for breast cancer between 1st January 2007 and 31th December 2017 in the region of southern Denmark (current population approximately 1.2 million). Follow up and assessments of all patients was conducted between 1st January 2019 and May 2020.
Recruitment	Baseline variables and data regarding breast cancer treatment were prospectively registered in the National Breast Cancer Registry from the Danish Breast Cancer Cooperative Group (DBCG), which was retrieved for this study. The DBCG include

	more than 95% of breast cancer patients in Denmark, and all breast cancer centers in Denmark follow the same treatment protocols regardless of geographical region. Identified patients were recruited in the outpatient clinic and sent questionnaires using REDCap.
Ethics oversight	This study was registered with the Danish Data Protection Agency (19/31965) and approved by The National Committee on Health Research Ethics (S-20180117) and The Danish Clinical Quality Program– National Clinical Registries (RKKP)/Danish Breast Cancer Cooperative Group (DBCG-2019-10-02)

Note that full information on the approval of the study protocol must also be provided in the manuscript.
